# Transcriptome analysis of multiple tissues reveals the potential mechanism of death under acute heat stress in chicken

**DOI:** 10.1186/s12864-023-09564-2

**Published:** 2023-08-16

**Authors:** Jiuhong Nan, Hongrui Yang, Li Rong, Zijia Jia, Sendong Yang, Shijun Li

**Affiliations:** 1 State Key Laboratory of Agricultural Animal Genetics, Breeding and Reproduction of Ministry of Education, College of Animal Science and Technology, Huazhong Agricultural University, Wuhan, 430070 China; 2 Hubei Hongshan Laboratory, Huazhong Agricultural University, Wuhan, 430070 China; 3 Key Laboratory of Smart Farming for Agricultural Animals, Ministry of Education, Huazhong Agricultural University, Wuhan, Hubei Province 430070 China

**Keywords:** Chicken, Acute heat stress, Death, Lung, Inflammation

## Abstract

**Background:**

Acute heat stress could induce high mortality and cause huge economic losses in the poultry industry. Although many studies have revealed heat stress-induced injuries of multiple tissues, the main target tissue and molecular mechanism of death under acute heat stress was largely unknown. This study systematically compared the transcriptome data of five main visceral tissues in chickens to reveal the response of multiple tissues to acute heat stress and determine the main target tissue of acute heat stress, further revealing the injuries of main target tissue and their potential mechanism by combing pathological section and qRT-PCR technologies.

**Results:**

The transcriptome data of five visceral tissues revealed that acute heat stress broadly caused inflammatory response and damaged tissues metabolic homeostasis. Among the five tested visceral tissues, the number of differentially expressed genes in the lung was the highest, and their fold changes were the greatest, indicating that the lung was the main target tissue of acute heat stress. The results of pathological section revealed severe inflammation, emphysema and pulmonary hemorrhage in the lung under acute heat stress. Our study found that some pro-inflammatory genes, including *CNTFR*, *FURIN*, *CCR6*, *LIFR* and *IL20RA*, were significantly up-regulated both in the heat-stress and heat-death groups, and their fold changes in the heat-death group were significantly greater than that in the heat-stress group. We also found an anti-inflammatory gene, *AvBD9*, exhibiting an extremely high expression in the heat-stress group but a low expression in the heat-death group.

**Conclusions:**

Our study found that acute heat stress caused multiple tissue injuries broadly and the lung was the main target tissue of acute heat stress in chicken. Acute heat stress caused a severe inflammatory response, emphysema, and pulmonary haemorrhage, The severe inflammatory response in the heat-death group was related to the up-regulation of pro-inflammatory genes and down-regulation of anti-inflammatory genes.

**Supplementary Information:**

The online version contains supplementary material available at 10.1186/s12864-023-09564-2.

## Introduction

With global warming, heat stress caused huge economic losses in the poultry industry [1]. According to the statistics, heat stress has caused losses of 125–165 million dollars to the poultry industry in the United States [2]. Heat stress is the sum of non-specific responses of chickens when they are under an excessively high temperature exceeding their thermoregulatory capacity, and can be roughly divided into acute and chronic heat stress according to the environmental temperature and exposure time. Under a high temperature, acute heat stress in chickens will be induced in a short time, causing high levels of tissue-damage and mortality [3]. Due to the influence of global warming, the occurrence frequency of extreme heat weather in various regions is increasing, which is short-lived but can still cause a high level of mortality. For example, in the summer of 2012, a large number of deaths of laying hens were caused by experiencing three periods of temperatures exceeding 38℃ in a short time [4]. Some regions, especially the tropical desert areas, have extremely high temperatures causing several problems for local poultry industry. For example, the highest temperature in the Cairo area of Egypt exceeded 44℃ for five years in the past decade (https://rp5.ru). Additionally, in modern poultry industry, temperature is controlled at the cost of a lot of energy, in which unexpected events such as power outages can’t be completely avoided. When power outages occurred, the environmental temperature would rise sharply. A reported example was that the environmental temperature was as much as 55 °C after a power outage and the mortality of population experienced such an event was as high as 98% [5].

In fact, a series of studies have proved that heat stress could cause injuries to multiple tissues in chickens. For example, heat stress could cause mitochondrial damage and produce excess reactive oxygen species (ROS) [6], thus resulting in the overwhelmed liver buffering system and oxidative injuries to enzymes, cellular lipids and mitochondrial membranes [7]. Heat stress could cause myocardial fiber rupture [8] and up-regulation of multiple genes involved in cardiac contraction [9]. Additionally, heat stress caused reduced layers and structural abnormalities of spermatogenic cells in chicken testis [10], and affected the expression of genes involved in metabolism and signal transduction [11]. Overal, heat stress could decrease growth, reproduction and immunity performance, thus causing huge enconmic losses [12]. However, there are no studies comparing the response of multiple tissues under acute heat stress to reveal the main target tissues of heat stress. The main target tissue and their corresponding molecular mechanism of death under acute heat stress were largely unknown.

RNA-Seq could determine the expression of all transcripts in a specific tissue at a certain time. Comparative analysis of transcriptome data could explain the expression changes under different treatments or stages. So far, RNA-Seq has been broadly used to study tissue injuries caused by environmental stress [13–15]. Thus, we selected heart, liver, spleen, lung and kidney to compare their transcript response to acute heat stress revealing the main target tissue of acute heat stress in chicken. Then, we integrated the results of the pathological section and transcriptome data to reveal the main injuries and their potential mechanism of the main target tissue. This study systematically explored the injuries of five visceral tissues under heat stress, preliminarily identifying the main target tissue of acute heat stress and its potential mechanism. Our study could deepen our understanding of death under acute heat stress and provide valuable reference materials for future prevention or treatment of acute heat stress.

## Results

### Transcriptome analysis reveals the response of five visceral tissues under acute heat stress

To examine the injuries of multiple tissues under acute heat stress systemically, we compared the transcriptome data of five visceral tissues, including heart, liver, spleen, lung and kidney, between the control and heat-stress groups. We found Q30 reads comprised more than 93% (Table S1), and PCA results based on the transcriptome data revealed that the samples of each tissue could be clustered respectively (Figure S1). To verify the transcriptome data, we randomly selected 11 differentially expressed genes (DEGs) to test their expression by qRT-PCR and found that the fold change correlation between RNA-Seq and qRT-PCR was 0.7003 (Figure S2), indicating that our transcriptome data was credible.

**Fig. 1:**
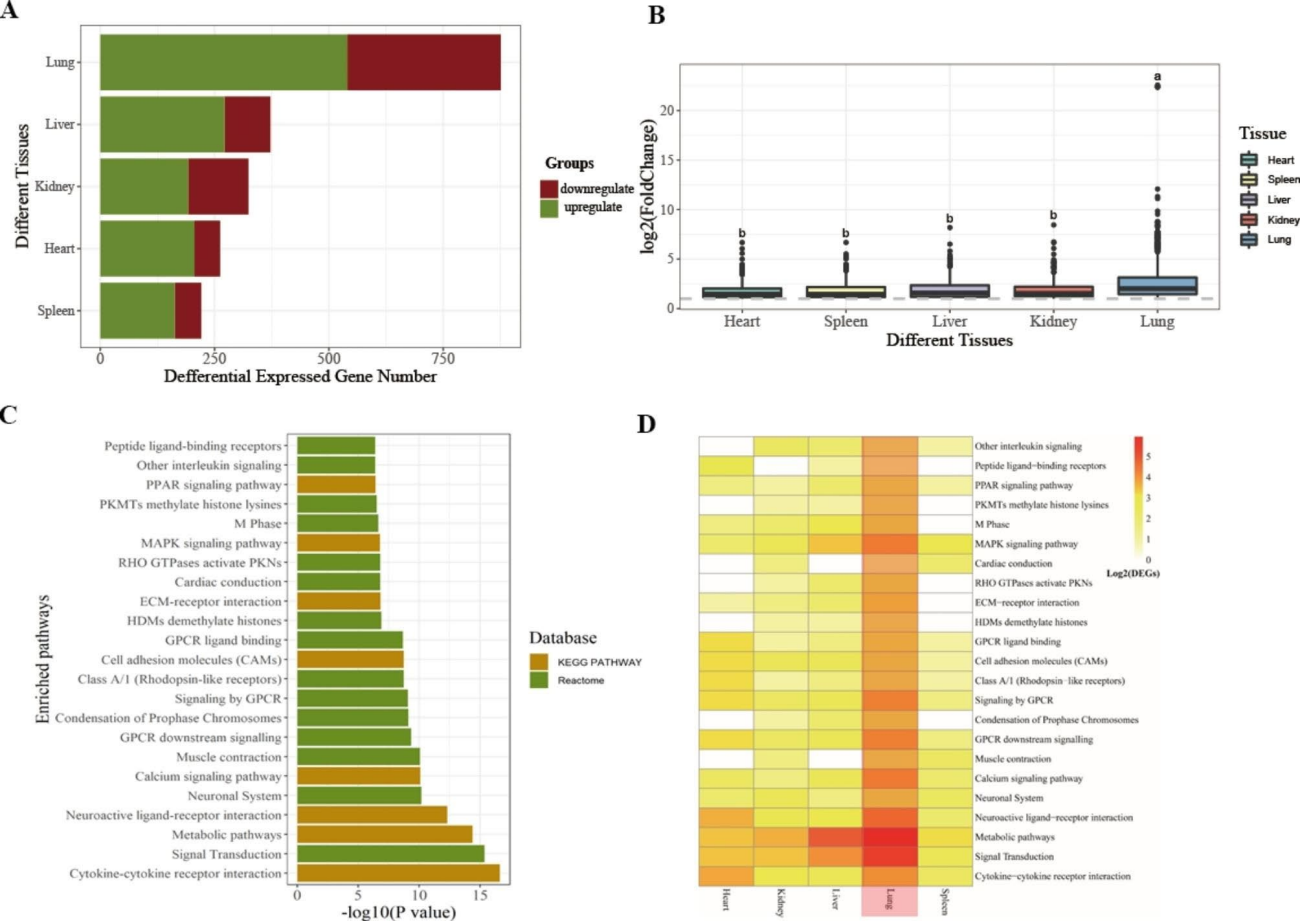
**Comparative analysis of five visceral tissues between the control and heat-stress groups.** (**A**) Summary of differentially expressed gene numbers in five visceral tissues between the control and heat-stress groups. (**B**) Distribution of fold changes of differentially expressed genes between the control and heat-stress groups. (**C**) Significant pathways enriched by differentially expressed genes between the control and heat-stress groups. (**D**) The number of differentially expressed genes from five visceral tissues involved in significant pathways

**Fig. 2:**
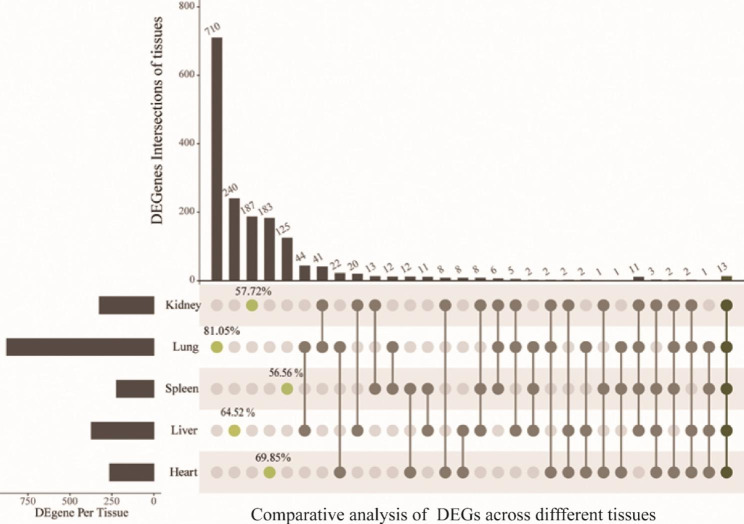
Comparative analysis of differentially expressed genes in different tissues between the control and heat-stress groups

A total of 1,697 DEGs were identified from the five visceral tissues, and the DEGs ranged from 221 to 876 in these five tissues, with the highest number in the lung (Fig. 1A). Additionally, the fold changes of DEGs in the lung between the control and heat-stress groups were significantly higher than those in other tissues (Fig. 1B). Besides, we found that responses to acute heat stress in different tissues exhibited high specificity at the gene level. The DEGs with tissue-specificity comprised more than 56% in every tissue (Fig. 2). There were 13 overlapping DEGs being identified in the five tissues, of which, eight genes were reported as members of the heat shock protein family (HSP), including *LOC107049075*, *DNAJA4*, *HSPA8*, *HSPA2*, *BAG3*, *HSP90AA1*, *HSPB9* and *LOC772158* (Table 1); three genes were transcribed from intronic regions of *HSPA8*, including *LOC112530276*, *LOC112530277* and *LOC112530278*; one gene was overlapped with *HSPH* (Table 1).

**Table 1:** The 13 overlapping differentially expressed genes in five visceral tissues between the control and heat-stress groups

Gene	Gene description	Fcunction	Log2(Fold Change)				
			Heart	Liver	Spleen	Lung	Kidney
LOC107049075	DnaJ heat shock protein family (Hsp40) member B1	related to HSP40	2.60	2.56	2.48	1.80	2.81
DNAJA4	DnaJ heat shock protein family (Hsp40) member A4		1.56	3.83	3.20	2.51	3.35
HSPA8	heat shock protein family A (Hsp70) member 8	related to HSP70	1.06	1.35	1.94	1.82	1.15
HSPA2	heat shock protein family A (Hsp70) member 2		2.89	5.82	3.08	2.25	3.29
BAG3	BAG cochaperone 3		1.46	3.68	3.57	3.38	3.57
HSP90AA1	heat shock protein 90 alpha family class A member 1	HSP90	1.14	1.61	2.17	1.64	1.69
HSPB9	heat shock protein family B (small) member 9	HSP9	4.13	8.18	6.67	6.59	6.09
LOC772158	heat shock protein 30 C-like	small heat shock protein 20 family	1.11	3.10	4.27	4.00	4.08
LOC112530277	small nucleolar RNA SNORD14	transcribed from intronic regions of the HSPA8 gene	2.51	3.59	2.57	2.10	3.29
LOC112530278	small nucleolar RNA SNORD14		2.29	3.78	2.61	2.62	3.03
LOC112530276	small nucleolar RNA SNORD14		2.96	3.90	3.22	2.21	3.35
HLA-F10AL1	class I histocompatibility antigen, F10 alpha chain-like 1, transcript variant X2	unknown	6.67	5.09	5.52	7.22	6.73
LOC107052088	uncharacterized LOC107052088	overlapping with HSPH1	1.87	3.81	3.41	1.48	1.56

Then, all DEGs were used for KEGG pathway enrichment analysis. The results showed that a total of 23 pathways exhibited significance, and 13 of these pathways, including cytokine-cytokine receptor interaction, signal transduction, metabolic pathway, neuronal system, and calcium signalling pathway, were shared in all tissues (Fig. 1C). These results indicated that acute heat stress could cause inflammatory responses broadly in every visceral tissue, and signal transduction and the nervous system might play an important regulatory role in response to acute heat stress, and metabolic homeostasis might also be remodelled under acute heat stress.

### Transcriptome data of multiple tissues reveals that the lung is the main target tissue under acute heat stress

As mentioned above, the DEG number in the lung was much higher than that in other tissues, and the fold changes of DEGs were significantly greater than that of other tissues. In the shared pathways, the number of DEGs involved in the GPCR signalling pathway from the lung was significantly higher than that of other tissues (Fig. 1D), indicating the signalling transduction in the lung under acute heat stress was more active. The number of DEGs enriched in neural ligand-receptor interaction in the lung was significantly higher than that in other tissues (Fig. 1D), indicating that the connection between lung and nervous system under acute heat stress was more intimate than that of other tissues. Besides, the number of DEGs in the lung related to the metabolic pathway was higher than that in other tissues (Fig. 1D), suggesting a greater effect on the lung metabolic homeostasis under acute heat stress. The number of DEGs in the heart and lung involved in cytokine-cytokine receptor interaction was higher than that in other tissues (Fig. 1D), indicating a more severe inflammatory response in the heart and lung under acute heat stress.

### Pathological section reveals severe inflammation and emphysema in the lung under acute heat stress

**Fig. 3:**
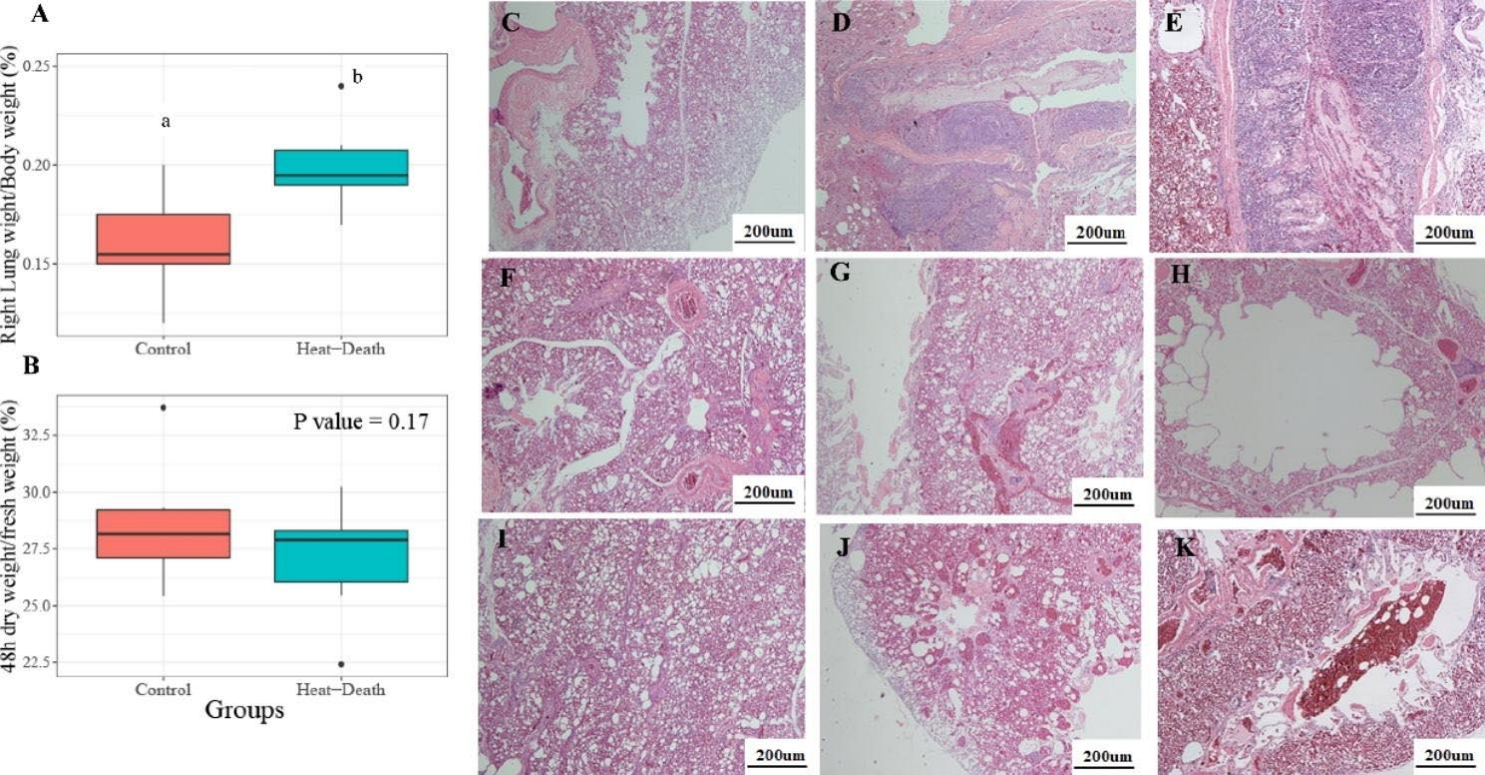
**Pathological findings in the lung under acute heat stress.** (**A**) the index of right lung weight divided by body weight between the control and heat-death groups. (**B**) the dry weight of right lung after drying at 37℃ for 48 h divided by its fresh weight. The different letters indicate significant difference between groups. (**C**-**K**) the representative photographs of HE-stained lungs, and their scale is 200 μm. (**C**) (**F**) and (I) show that pathological lung sections in the control group were normal. Severe inflammation was observed in the heat-stress group (**D**) and heat-death group (**E**). Emphysema was observed in the heat-stress group (**G**) and heat-death group (**H**), and alveolar ruptures were observed in the heat-death group (**H**). Pulmonary hemorrhage was observed in the heat-stress group (**J**) and heat-death group (**K**)

Additionally, we set heat-stress and heat-death groups to examine the lung injuries under acute heat stress. The heat-death time of these individuals ranged from 49 to 75 min and we randomly selected three individuals for further analysis with their heat-death time from 53 to 75 min (Table S3). We found the right lung index of the heat-death group was significantly higher than that of the control group (P < 0.01) (Fig. 3A), and the ratio between right lung weight after drying at 37℃ for 48 h and fresh weight of right lung in the heat-death group was lower than that of the control group (P = 0.17) (Fig. 3B). Compared to the control group (Fig. 3C, F, I), we detected excessive inflammatory cell infiltration in the lung epithelial mucosa of the heat-stress group (Fig. 3D) and heat-death group (Fig. 3E), mild emphysema in heat-stress group (Fig. 3G), severe emphysema and alveolar rupture in heat-death group (Fig. 3H), pulmonary haemorrhage in both groups (Fig. 3J and K). In short, our results revealed that acute heat stress could damage the normal structure of the lung, and cause severe inflammatory response, emphysema and pulmonary hemorrhage.

### Evidence reveals the potential mechanism of inflammation in the lung under acute heat stress

**Fig. 4:**
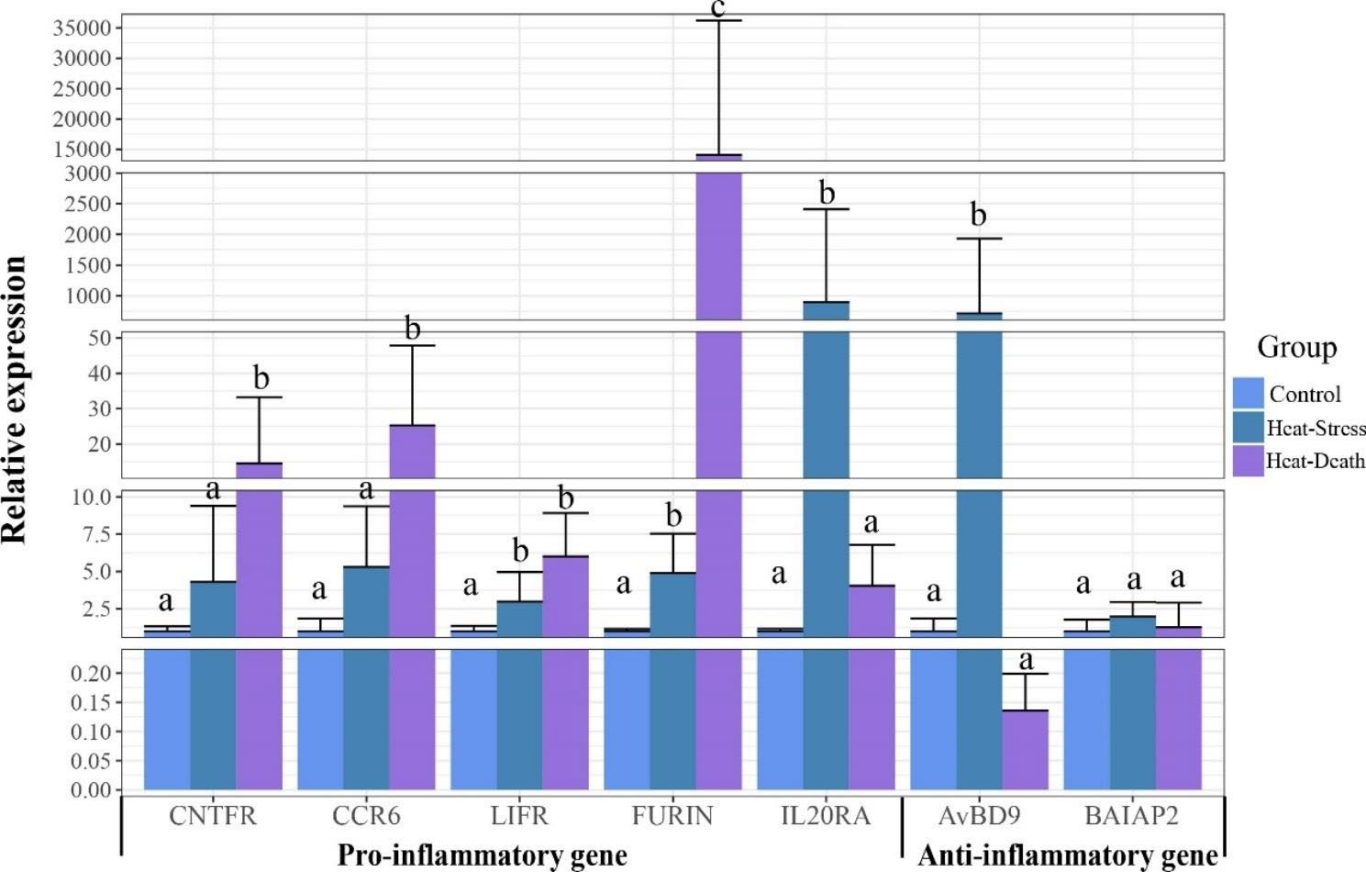
**Relative expression level of several inflammatory genes by qRT-PCR.** Among these genes, *CNTFR*, *CCR6*, *LIFR*, *FURIN* and *IL20RA* belong to pro-inflammatory genes, and *AvBD9* and *BAIAP2* belong to anti-inflammatory genes. Expression levels were normalized against that of the *GAPDH* gene. Values are means (n = 3) and error bars represent the standard deviation. Different letters above the bars indicate a significant difference in different groups

A total of 10 genes involved in cytokine-cytokine receptor interaction were differentially expressed in the heat-stress group, including *BMP10*, *IL20RA*, *GDF2*, *GDF15*, *THPO*, *CNTFR*, *INHBA*, *CCR6*, *LIFR* and *BMP3* (Figure S3). In these 10 genes, all genes were up-regulated significantly in the heat-stress group except *CCR6*, *LIFR* and *BMP3*. *CCR6*, *LIFR*, *IL20RA* and *CNTFR* were selected to examine their expression in the heat-stress and heat-death groups because they have been reported to be pro-inflammatory genes. Besides, the other 3 genes, including *FURIN*, *AvBD9* and *BAIAP9*, were also targeted for validation because they were reported to be related to the innate immune system. In these 7 genes used for validation, the expression trends of five genes in transcriptome data were consistent with the results of qRT-PCR (Table S2). Transcriptome data revealed that *CCR6* and *LIFR* were significantly down-regulated with low-fold changes in the heat-stress group (Table S2). The results of qRT-PCR revealed their up-regulation, suggesting that we should be particularly careful about DEGs with low fold change when using the transcriptome data. All these genes for validation exhibited up-regulations in the heat-stress group, including 5 pro-inflammatory genes and 2 anti-inflammatory genes (Fig. 4). The 4 pro-inflammatory genes, including *CNTFR*, *CCR6*, *LIFR* and *FURIN*, were up-regulated both in the heat-stress and heat-death groups, with higher fold changes in the heat-death group than in the heat-stress group. The pro-inflammatory gene *IL20RA* also exhibited up-regulation in both groups, but its fold change in the heat-death group was lower than that in the heat-stress group. The anti-inflammatory gene *AvBD9* exhibited significant up-regulation in the heat-stress group but down-regulation in the heat-death group with potential significance. Another anti-inflammatory gene *BAIAP2* exhibited insignificant up-regulation in the heat-stress and heat-death groups.

## Discussion

Chicken has no sweat glands and is covered with feathers, thus chicken has a higher temperature than mammals. Thus, chickens are more susceptible to heat stress than mammals when under high temperatures. Many previous studies have found that heat stress could induce multiple tissue injuries [6, 7, 16, 17], and this study found that heat treatment with a high temperature in a short time could cause death in chickens. However, the individual differences in heat tolerance still existed in the pure laying hens with highly similar genetic background and feeding in the same environment. In this study, we defined the individuals with 50 min heat exposure as the heat-stress group, which is not the most perfect solution because of the individual difference in physiological response to the same heat treatment even though in the same pure populations. Thus, the study of physiological indicators accurately assessing the degree of heat stress is necessary in the future. Recently, several studies about predicting the heat stress damage have been published. A study thought that heat stress could damage intestinal integrity and microbiota, and thus serum FD4, d-lactate and diamine oxidase might be used for predicting the heat stress damage [18, 19]. Based on transcriptome data, we found acute heat stress-induced inflammatory response broadly in five main visceral tissues, especially in the heart and lung. We speculated that the inflammatory response in the heart and lung were more severe than that in other tissues because evaporating water by breathing was a decisive way for heat loss in chickens under heat stress, in which the heart and lung acted as the breathing center and power tissue, respectively.

This study and previous studies have shown that the metabolic homeostasis of multiple-tissue was affected under heat stress [20, 21], which may be related to excess load heat for dissipation under heat stress. Also, our study found that many differentially expressed genes in the heat-stress group were involved in multiple-pathway related to the neuronal system and signalling transduction, including Neuroactive ligand-receptor interaction, neuronal system, signalling transduction, GPCR signalling pathway and Calcium signalling pathway, suggesting the important regulatory role of the neuronal system in response to acute heat stress.

A key finding of this study was that acute heat stress induced a stronger response in the lung than in other visceral tissues, causing a severe inflammation in the lung. Pathological section revealed that acute heat stress induced severe lung injuries, including severe inflammatory response, emphysema and pulmonary haemorrhage, and lung failure syndrome appeared in the heat-death group. Acute heat stress could cause heat stroke in humans with high levels of mortality. Clinical data showed that acute heat stress caused lung injuries in humans, mainly characterized by pulmonary oedema and acute respiratory distress syndrome, and was one of the important causes of death from heat stroke in humans [22]. It should be noted that the pressure on the respiratory system for losing heating chickens is much higher than that in mammals. Thus, we speculated that lung failure syndrome was an important cause of death under acute heat stress. The previous studies rarely focused on the response of lung to acute heat stress in chicken, our study provided strong evidence for the important role of lung in death under acute heat stress.

Our study found that acute heat stress caused emphysema and pulmonary haemorrhage, and TGFβ plays an important role in the maintenance of basic lung structure. Previous studies showed that the abnormal expression of *TGFβ* could cause emphysema symptoms [23], and this study found that the symptoms of emphysema might be related to the differential expression of multiple genes in the TGFβ family. For example, *GDF15* exhibited a higher expression in the heat-stress group and was reported to be related to airway obstruction and emphysema parameters. In the same cigarette exposure, the inflammatory response of the lung in a *GDF15*-knockout group was significantly lower than that in the control group [24]. Thus, we speculated that up-regulation of *GDF15* might promote emphysema under acute heat stress. Another gene, *INHBA*, which belonged to TGFβ, was reported to promote the inflammatory response in the lung [25] and exhibited a significantly higher expression in the heat-stress group. Additionally, it’s reported that the proteins of the integrin family were closely related to the function of TGFβ. As a classical example, *ITGB6* in the integrin family was reported to affect acute lung injury and emphysema by an *ITGB6*-knockout assay [26], and *ITGB6* exhibited a higher expression in the heat-stress group in this study. Thus, we speculated that the differential expressions of some genes in TGFβ and integrin family were related to pulmonary emphysema under acute heat stress.

Traditionally, inflammation was considered a defensive response to infection or injuries. A recent study indicated that inflammation could be caused by tissue stress and dysfunction in the absence of infection and tissue injuries [27]. Our study found severe inflammation in the lung under heat stress might be related to the differential expressions of multiple genes involved in the cytokine-cytokine receptor interaction and innate immune system. For example, *CNTFR*, *FURIN*, *CCR6*, and *LIFR*, reported as pro-inflammatory genes, were significantly up-regulated in the heat-stress group, and their fold changes increased significantly as the heat treatment time prolonged. Among these four genes, *CNTFR* belonging to IL6 family was essential in inflammatory response, metabolism and tissue regeneration, and their abnormal expressions wildly existed in cancer and autoimmune diseases [28]. The protein encoded by *FURIN* was an important modulator of T-cell-dependent adaptive immunity [29]. Our study found that *FURIN* exhibited a higher expression both in the heat-stress and heat-death groups than that in control group, and the fold change of heat-death group was much higher than that of heat-stress group, suggesting that heat stress-induced T-cell-dependent adaptive immune response by up-regulating this gene, but more studies were needed. *CCR6* is a chemokine receptor and is expressed in immature dendritic, B lymphocytes and memory T cells [30], the up-regulation of *CCR6* suggested the increasing immune response with heat stress time prolonging. LIFR protein was encoded by the *LIFR* gene, and its high expression through an autocrine manner could promote the production of IL-6 and a range of other inflammatory genes [31]. *IL20RA* encoding α subunit of IL20 receptor exhibited higher expressions both in the heat-stress and heat-death groups than that in control group, and could regulate the expression of inflammatory mediators by regulating the JAK-STATs pathway [32]. Our study observed the up-regulation of these pro-inflammation genes under acute heat stress, revealing the potential mechanism of immune response to heat stress at the expression level. *AvBD9* was an anti-inflammatory gene, and the AvBD9 protein, as a natural antibacterial peptide, could effectively inhibit or kill the gram-positive and negative bacteria, mycoplasma, spirochaeta, fungi, viruses, protozoa, and film to resist the invasion of pathogenic microorganisms [33]. This study observed an extremely high expression of *AvBD9* in the heat-stress group and a low expression in heat-death group. To sum up, some pro-inflammatory genes and a small amount of anti-inflammatory genes were significantly up-regulated in the heat-stress group trying to maintain the balance of inflammatory response. With the heat treatment time increasing, the expressions of pro-inflammatory genes increased, but the expression of anti-inflammatory genes decreased, thus causing severe inflammation in the heat-death group. Overall, our study deepens our understanding of the mechanism of death under acute heat stress, but more systematic and in-depth studies are still needed in future.

## Conclusions

In this study, we compared and analyzed the transcriptome data of five visceral tissues between the control and heat-tress groups, revealing that acute heat stress could induce inflammation responses broadly in the five tissues and damage metabolic homeostasis. Among five visceral tissues, the response of the lung under acute heat stress was much higher than that of other tissues. We observed severe inflammation, emphysema and pulmonary haemorrhage in the lung under acute heat stress, and found that the differential expressions of multiple pro-inflammatory and anti-inflammatory genes might be related to the severe inflammation in the lung under acute heat stress. This study systemically suggested that the lung was an important target tissue of acute heat stress in chickens, and preliminarily revealed the potential mechanism of lung injuries. It helps deppen our understanding of the mechanism of death under acute heat stress and provides valuable materials for reducing the damage and mortality under acute heat stress.

## Materials and methods

### Sampling, treatment and grouping

In this study, the 18 healthy, adult white leghorn laying hens were divided into the control, heat-stress and heat-death groups. It’s worth noting that these birds have been raised in the same chicken coop with the same nutrient level, and diets met or exceeded the nutritional requirement stated in NRC (1994) since birth. The control group was treated at 25 ± 1℃ with a relative humidity of 35 ± 5% for 50 min. The heat-stress group was treated at 45 ± 1℃ with a relative humidity of 35 ± 5% for 50 min. To explore the main cause of death caused by acute heat stress, we set a heat-death group in this study. The heat-death group was treated in the environment of 45 ± 1℃ with a relative humidity of 35 ± 5% close to death but not dead. All samples after treatment were stunned by electrical stunning and then slaughtered with a quick, exsanguination by severing the carotid artery. In this study, two copies of the heart, liver, spleen, lung and kidney were collected. One copy was homogenized with 1ml TRIzol reagent (ambion by life technologies, USA.) for RNA extraction, and the other copy was put into 4% paraformaldehyde (National Pharmaceutical Group Chemical Reagent Co., Ltd., China) for the histopathological examination.

### Total RNA extraction, RNA-Seq and DEGs identification

To examine the tissue injuries under heat stress, 3 samples of the control and heat-stress groups were randomly selected to perform RNA-Seq. Total RNA was extracted by TRIzol reagent based on the manufacturer’s instruction, whose quality was examined by NanoDrop and Agilent 2100. The mRNA was isolated by Oligo (dT)-attached magnetic beads, and randomly fragmented in a fragmentation buffer. First-strand cDNA was synthesized with the fragmented mRNA as a template and random hexamers as primers, followed by second-strand synthesis with the addition of PCR buffer, dNTPs, RNase H and DNA polymerase I, and cDNA was purified with AMPure XP beads. Double-strand cDNA was subjected to the end repair, and a cDNA library was obtained by certain rounds of PCR. A total of 36 libraries were sequenced on Illumina HiSeq 2500 platform (Illumina Inc., San Diego, USA) according to Illumina’s RNA-Seq instructions to obtain 150-bp paired-end reads for further study.

The low-quality reads and adapters were removed with Trimmomatic-0.39 (Bolger, et al., 2014). High-quality reads were aligned to the reference genome (GRCg6a) by hisat2 [34], and gene count was generated by HTSeq [35]. Differentially expressed genes (DEGs) were detected by DESeq2 package [36]. The genes with P-value < 0.05 and |Log2 (fold change)| >1 were defined as DEGs. DEGs were used to examine their enriched pathways with KOBAS 3.0 [37]. For the most important pathways, the raw images were obtained from KEGG [38–40], and the related significant gene information was highlighted using Pathview[41].

### Histopathological examination

The samples fixed in 4% formaldehyde solution (National Pharmaceutical Group Chemical Reagent Co., Ltd., China) were paraffin-embedded, and 3 μm-thick sections of the paraffin embedding samples were obtained and used for hematoxylin-eosin (H&E) staining. We examined all the histological slides with an Olympus light microscope and digitized images with a Nikon DS-U3 camera (Olympus Corporation, Tokyo, Japan) control unit connected to a Nikon Eclipse CI upright optical microscope.

### Quantitative real-time PCR (qRT-PCR)

We performed qRT-PCR technology with 3 samples of the control, heat-stress and heat-death groups to quantify the expressions of key genes and verify the reliability of RNA-Seq. Total RNA from every sample was converted into cDNA using HiScript Reverse Transcriptase (Vazyme Biotech Co., Ltd., Nanjing, China), and cDNA was used for qRT-PCR with GAPDH as the internal control gene. qRT-PCR was performed with SYBR Green I kit (Vazyme Biotech Co., Ltd., Nanjing, China), with 1.6ul cDNA and 0.2ul of forward and reversed primers in a final volume of 10ul. The samples were centrifuged briefly and run on the PCR machine using the program ( 95℃ for 5 min, 95℃ for 30s, 58℃ for 30s, 72℃ for 30s, 72℃ for 5 min and 25℃ for 1 min) in triplicate. The relative gene expression levels were determined using the 2^–ΔΔCt^ method, the mean value of three technological replications was used to represent the expression of the individual and three biological replications of different groups was used to estimate the difference in different groups. We used LSD.test function to estimate the difference in gene expression in different groups. The primer sequences of genes for validation are represented in Table 2.

**Table 2:** Primer sequences used in qRT-PCR

Gene	Forward primer sequence (5’-3’)	Reverse primer sequence (5’-3’)	Product length (bp)
IL20RA	GGAGGAGAAGTCCTGATGGA	GGCGTAGTGACATGGATCTG	193
LIFR	TGCCTGGAACATGGACACTC	GGAAGCAGAAACTGTCGGGA	108
CNTFR	GGCACGAGAGAGATGGTCTG	ATGGCGAACTCGTCAAAGGT	161
FURIN	CTACGGCACACTGACCAAGT	TTCAGGCAGCTCTTCTGGTG	165
CCR6	GGGGGCTTCATTTGCATACC	ACGATGTTGCACGACTCTG	195
AvBD9	AGCAGAGGACAATCATGAGAA	CACGGCAGGTCCCAATGTCAA	177
ACTC1	AGAGCTACGAATTGCCTGATG	TAGTTTCATGAATACCAGCAG	120
CACNAD	CAATTGTGTGGCCTTAGCTGT	CATTTCTAACATACGCATTGG	173
FBP2	GTCGAACTCCCTGGTGATCAA	CATCCAGAGGGTCGAAGCACA	137
PDE4B	GTACATCTCCAACACCTTCCT	TGCTGGTATTATTTAAGCTGG	155
BAIAP2	GTTTCCTTTCTCCTACACACG	AGGTGTTGACGTTCTGCGATG	179

### Electronic supplementary material

Below is the link to the electronic supplementary material.

Supplementary Material 1

## Data Availability

Sequencing data in this study are submitted to the Genome Sequence Archive in BIG Data Center under the BioProject: PRJCA010811 (https://ngdc.cncb.ac.cn/bioproject/browse/PRJCA010811).

## Abbreviations

DEG: Differentail expressed gene
HE: Hematoxylin-eosin staining
qRT-PCR: Quantitative real-time PCR
PCA: Principle component analysis
HSP: Heat shock protein
DNAJA4: DnaJ heat shock protein family (Hsp40) member A4
HSPA8: Heat shock protein family A (Hsp70) member 8
HSPA2: Heat shock protein family A (Hsp70) member 2
BAG3: BAG cochaperone 3
HSP90AA1: Heat shock protein 90 alpha family class A member 1
HSPB9: Heat shock protein family B (small) member 9
IL20RA: Interleukin 20 receptor subunit alpha
GDF2: Growth differentiation factor 2
GDF15: Growth differentiation factor 15
THPO: Thrombopoietin
CNTFR: Ciliary neurotrophic factor receptor
INHBA: Inhibin subunit beta A
CCR6: C-C motif chemokine receptor 6
LIFR: LIF receptor subunit alpha
BMP3: Bone morphogenetic protein 3
FURIN: Furin, paired basic amino acid cleaving enzyme
AvBD9: Avian beta-defensin 9
BAIAP2: BAR/IMD domain containing adaptor protein 2
TGFβ: Transforming growth factor beta family
