# Supplementary material for: Transcriptome analysis of multiple tissues reveals the potential mechanism of death under acute heat stress in chicken
Source: BMC Genomics. 2023 Aug 16;24:459. doi: 10.1186/s12864-023-09564-2 (PMC10429076; doi:10.1186/s12864-023-09564-2)
Supplement: Supplementary file 1 — Supplementary Material 1 [file 12864_2023_9564_MOESM1_ESM.pdf]

Table S1 Summary of all RNA-Seq libraries

Sample ID	Groups	Tissues	Obtained Reads	Obtained Base(bp)	Q20(%)	Q30(%)	GC(%)
BBX1	Control	heart	24,365,648	7,243,769,182	97.85	94.06	49.5
BBX2	Control	heart	19,999,806	5,980,884,880	97.86	93.86	47.79
BBX3	Control	heart	23,889,360	7,095,117,388	97.73	93.77	49.41
BBG1	Control	liver	23,105,179	6,876,476,988	98.13	94.69	49.56
BBG2	Control	liver	23,065,439	6,850,993,600	98.19	94.78	49.38
BBG3	Control	liver	25,182,274	7,485,645,394	98.09	94.52	48.99
BBP1	Control	spleen	23,277,302	6,926,601,378	97.89	94.34	50.87
BBP2	Control	spleen	22,240,902	6,602,166,632	97.98	94.51	50.36
BBP3	Control	spleen	23,137,449	6,879,505,942	97.79	94.08	50.07
BBF1	Control	lung	21,554,549	6,436,083,620	97.91	94.1	47.45
BBF2	Control	lung	21,039,121	6,257,070,096	97.92	94.39	50.34
BBF3	Control	lung	26,500,933	7,882,219,208	97.96	94.43	50.22
BBS1	Control	kidney	27,072,407	8,068,234,800	97.48	93.21	49.67
BBS2	Control	kidney	26,402,972	7,846,912,424	97.79	93.84	50.1
BBS3	Control	kidney	27,856,961	8,304,809,296	97.83	93.84	49.9
BHX1	Heat-stress	heart	20,918,317	6,215,138,354	97.8	94.04	49.68
BHX2	Heat-stress	heart	28,689,145	8,521,218,418	97.91	93.97	49.62
BHX3	Heat-stress	heart	21,792,638	6,484,485,590	97.71	93.79	49.64
BHG1	Heat-stress	liver	24,220,159	7,207,671,100	97.88	94.15	49.22
BHG2	Heat-stress	liver	21,065,229	6,266,647,780	97.95	94.34	49.43
BHG3	Heat-stress	liver	19,431,342	5,783,021,994	98.01	94.44	49.32
BHP1	Heat-stress	spleen	21,106,237	6,259,113,976	97.71	93.98	50.05
BHP2	Heat-stress	spleen	21,336,869	6,349,534,718	97.6	93.77	50.96
BHP3	Heat-stress	spleen	20,843,318	6,189,380,420	97.66	93.88	50.5
BHF1	Heat-stress	lung	21,584,085	6,431,921,970	97.78	94.1	50.4
BHF2	Heat-stress	lung	19,175,671	5,707,575,758	97.59	93.74	51.2
BHF3	Heat-stress	lung	21,319,561	6,343,103,792	97.93	94.2	48.7
BHS1	Heat-stress	kidney	19,193,665	5,720,526,734	97.71	93.86	49.76

BHS2	Heat-stress	kidney	21,098,454	6,275,422,106	97.8	94.1	50.25
BHS3	Heat-stress	kidney	19,653,909	5,852,298,368	97.63	93.72	49.89

Table S2 Fold changes of key genes between the control and heat-stress groups in the lung from RNA-Seq and qRT-PCR

**Gene**	**Log2FC(qPCR)**	**Log2FC(RNA-Seq)**
FURIN	2.29	1.66
CCR6	2.41	-1.5
LIFR	1.57	-1.9
CNTFR	2.1	2.75
AvBD9	9.48	12.07
BAIAP2	2.06	2.58
ACTC1	-1.14	-2.93
CACNAD	0.29	-1.05
FBP2	3.29	4.15
PDE4B	1.35	1.1
IL20RA	9.82	5.02

Table S3 Summarized data about heat-death group

Sample ID	Groups	Body weight (Kg)	Rectal temperature before heat stress (℃)	Rectal temperature after heat stress (℃)	Heat-death time (min)
BJ1	Control	1.69	40.8	41	/
BJ2	Control	1.79	40	40.5	/
BJ3	Control	1.87	40.9	40.8	/
**SJ2**	**heat-death**	**1.61**	**41**	**46.8**	**75**
**SJ4**	**heat-death**	**1.74**	**41**	**46.6**	**53**
**SJ5**	**heat-death**	**1.76**	**41.5**	**46**	**62**
SJ1	heat-death	1.67	41	46.8	49
SJ3	heat-death	1.74	41	46.6	51
SJ6	heat-death	1.7	41	45.4	62

Note: The samples of heat-death group using for qPCR were marked in bold.

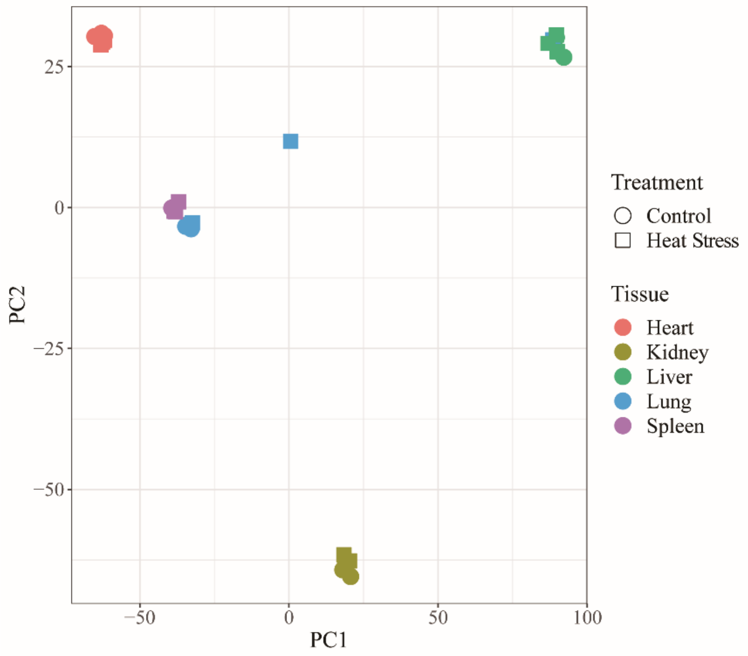

Figure S1 Principal component analysis of five visceral tissues between the control and heat-stress groups based on transcriptome data. The samples of different treatment were marked in different shapes and that of different tissues were marked in different colors.

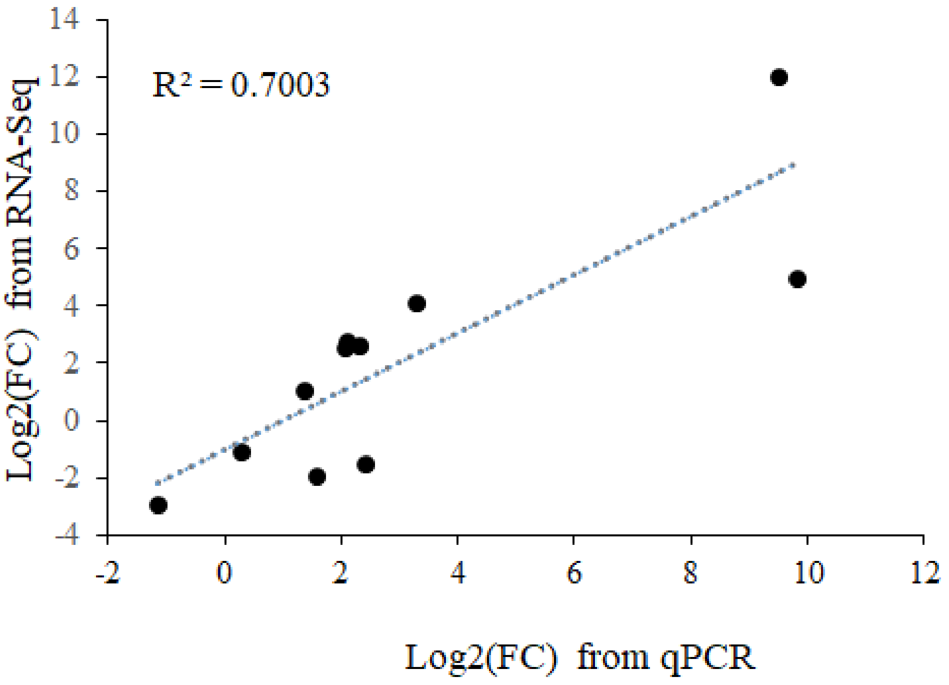

Figure S2 The correlation of log2 (FC) from RNA-Seq and qRT-PCR

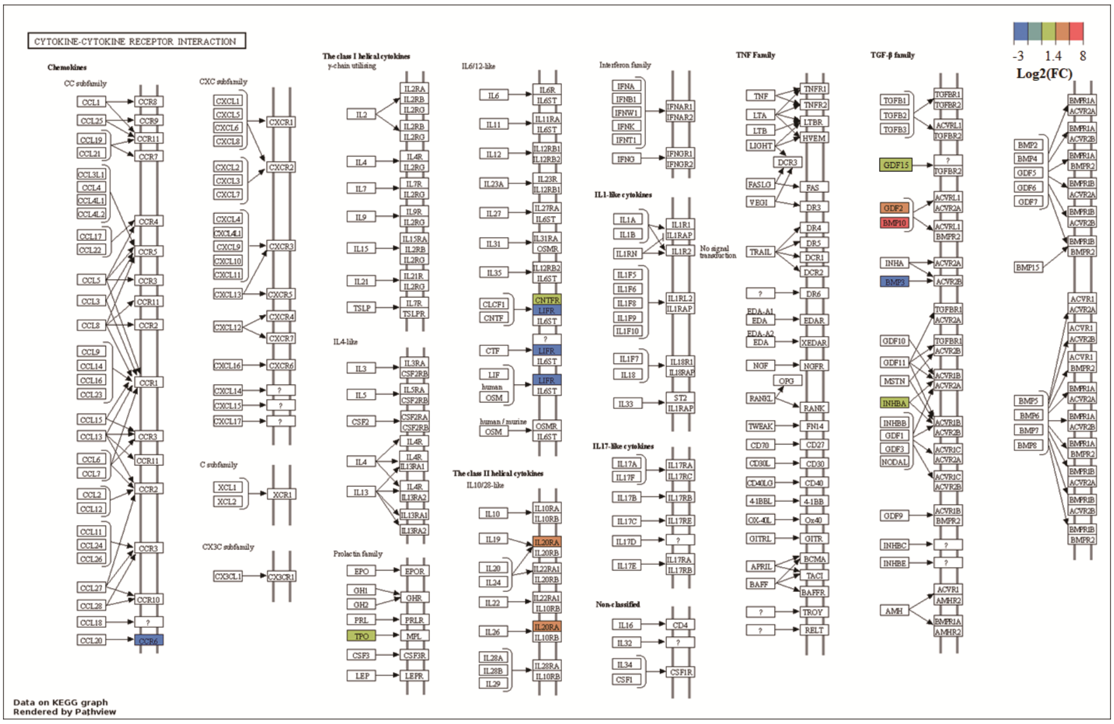

Figure S3 Differential expressed genes under heat stress involved in cytokine-cytokine receptor interaction. Differential expressed genes were highlighted, those fold changes between control and heat-stress groups were marked in different colors.
